# EARLY DETECTION OF POOR ADHERENCE TO TREATMENT OF PEDIATRIC RHEUMATIC DISEASES: PEDIATRIC RHEUMATOLOGY ADHERENCE QUESTIONNAIRE - A PILOT STUDY

**DOI:** 10.1590/1984-0462/;2019;37;2;00015

**Published:** 2019-03-18

**Authors:** Vanessa Bugni Miotto e Silva, Karine Yoshiye Kajiyama Okamoto, Luciana da Silva Ozaki, Claudio Arnaldo Len, Maria Teresa de Sande e Lemos Ramos Ascensão Terreri

**Affiliations:** a Escola Paulista de Medicina, Universidade Federal de São Paulo, São Paulo, SP, Brazil.

**Keywords:** Rheumatology, Pediatrics, Medication adherence, Patient compliance, Surveys and questionnaires, Reumatologia, Pediatria, Adesão à medicação, Cooperação do paciente, Inquéritos e questionários

## Abstract

**Objective:**

To develop a questionnaire that allows the early detection of patients at risk for poor adherence to medical and non-medical treatment in children and adolescents with chronic rheumatic diseases.

**Methods:**

The Pediatric Rheumatology Adherence Questionnaire (PRAQ) was applied in recently diagnosed patients within a period of one to four months after confirmation of the rheumatic disease. After six months, the patients’ adherence to the medical and non-medical treatment was assessed. An internal consistency analysis was conducted to eliminate redundant questions in the PRAQ.

**Results:**

A total of 33 patients were included in the pilot study. Six months after the PRAQ had been applied, poor global adherence was observed in seven (21.2%) patients and poor adherence to medical treatment in eight (24.2%) patients. No correlation was observed between the PRAQ scores and the percentages of adherence, as well as the stratification for each index, except for a tendency to a correlation between socioeconomic index and poor adherence to medical treatment (p=0.08). A new PRAQ questionnaire with 25 of the 46 original questions was generated as a result of the reliability analysis.

**Conclusions:**

The usefulness of this questionnaire in clinical practice should be still evaluated. Due to the importance of a tool for the early detection of rheumatic patients at risk of poor adherence to treatment, the new PRAQ questionnaire should be reviewed and applied in a larger study to better define its validity and reliability.

## RESUMO

**Objetivo:**

Desenvolver um questionário que permitisse a detecção precoce de pacientes em risco de má adesão ao tratamento medicamentoso e não medicamentoso para crianças e adolescentes com doenças reumáticas crônicas.

**Métodos:**

O Questionário de Adesão em Reumatologia Pediátrica (QARP) foi aplicado em pacientes recentemente diagnosticados, de um a quatro meses após a confirmação da doença reumática. Seis meses depois, foi avaliada a adesão do paciente ao tratamento medicamentoso e não medicamentoso. Foi realizada uma análise de consistência interna para eliminar questões redundantes no QARP.

**Resultados:**

Ao todo, 33 pacientes foram incluídos no estudo-piloto. Seis meses após a aplicação do QARP, observou-se baixa adesão global em sete pacientes (21,2%) e baixa adesão ao tratamento medicamentoso em oito (24,2%) pacientes. Não foi encontrada correlação entre os escores QARP e as porcentagens de adesão, bem como a estratificação para cada indicador, com exceção de uma tendência a uma correlação entre o indicador socioeconômico e a baixa adesão ao tratamento medicamentoso (p=0,08). Um novo questionário QARP com 25 das 46 perguntas originais foi gerado como resultado da análise de confiabilidade.

**Conclusões:**

A utilidade deste questionário na prática clínica ainda deve ser avaliada. Devido à importância de uma ferramenta para a detecção precoce de pacientes reumáticos em risco de má adesão ao tratamento, o novo questionário QARP deve ser revisado e aplicado em um estudo maior para que sua validade e confiabilidade sejam mais bem definidas.

## INTRODUCTION

Interest in the assessment of the adherence of children and adolescents to the treatment of chronic diseases has increased over the last three decades. This is particularly due to the harmful, costly, and irreversible effects that may occur in the case of treatment failure.^1^ ^,^ ^2^

The primary objectives of studies assessing adherence to the treatment of chronic diseases are predominantly the evaluation of difficulties and the development of strategies that promote stricter adherence, including patient information, therapeutic management, and environmental changes.^1^ ^,^ ^3^

Among other chronic diseases, pediatric rheumatic diseases are characterized by a high morbidity and mortality rate, particularly in cases of treatment failure. Overall, treatment is time-consuming and it requires a multidisciplinary interaction, the daily use of multiple drugs, and physical therapy with the potential use of orthosis.

Therefore, a continuous monitoring of the patient is required. As a result, treatment is a complex process that is hard to be adhered to by patients and caregivers.^4^ Issues most commonly related to poor adherence are demographic, which is particularly true in relation to the age of the patient, and the specificity of the individual disease. In addition, psychological, sociocultural, economic, and family-related factors are discussed.^1^ ^,^ ^3^ ^,^ ^4^ ^,^ ^5^ The early detection of these issues for each individual suffering from a rheumatic disease contributes to the development of an effective strategy to promote adherence, improve the quality of life, and facilitate a long-term prognosis.^6^ ^,^ ^7^

However, early detection of these risk factors is a difficult task in daily practice since medical anamnesis is mainly focused on clinical aspects and does not include details that may indicate a risk of poor adherence to the treatment. Therefore, the goal of this study was to develop a questionnaire that allows the early detection of patients at risk for poor adherence to medical and non-medical treatment in children and adolescents with chronic rheumatic diseases.

## METHOD

Patients from an outpatient Pediatric Rheumatology clinic aged between two and 18 years and with a recent diagnosis time of up to four months were consecutively included in the study. All patients met the applicable diagnostic or classification criteria for juvenile idiopathic arthritis (JIA), systemic lupus erythematosus, dermatomyositis, or localized scleroderma.^8^ ^,^ ^9^ ^,^ ^10^ ^,^ ^11^ ^,^ ^12^ All patients had to be taking at least one prescribed medication at the time of the evaluation.

The questionnaire entitled “Pediatric Rheumatology Adherence Questionnaire” (PRAQ) was adopted for the data analysis. Its development was structured in five steps:

- Set-up of a panel of caregivers and professionals (physicians, physical therapists, and psychologists).
- Development of a set of suitable questions to analyze the adherence to the treatment (each caregiver and professional came up with 10 to 20 relevant items).
- Validation and limitation of the relevant questions through a rating scale (1 - barely relevant and 5 - very relevant) by the same panel of caregivers and professionals.
- Organization of the questions in theme groups and elimination of redundant questions to comply with the understanding of items and to prevent bias.
- Conduction of a pretest of comprehension with the caregivers to adjust the final template for the questionnaire.

A total of 105 questions were relevant to analyze adherence to the treatment. After an analysis of redundancy conducted by the panel members (four physicians, two physical therapists, one psychologist and two caregivers), 51 questions remained. Then, based on a pretest of comprehension conducted with the caregivers, 46 questions were selected for the final template of the PRAQ.

The PRAQ questions were divided into five blocks with the following indexes:

- Socioeconomic status.
- Relationship with the healthcare team and system.
- Health status.
- Treatment.
- Caregiver/patient relationship.

The questionnaire was applied to the caregivers by two evaluators who were not participating in the regular patient treatment. For each question, the following three answers were possible: “yes”, “no”, and “not applicable”. For each answer “yes”, a point is added, and the higher the final score of the questionnaire the greater the risk of poor adherence was hypothesized.

The PRAQ was only applied to the caregivers within a period of one to four months after the disease diagnosis. Six months after the PRAQ application, the adherence of the patients was assessed by a quantitative questionnaire previously developed that evaluates the following three items^4^:

- Demographic status, clinical, and laboratory data.
- Adherence to the medical treatment over the last four weeks.
- Attendance of visits and performance of exams in the last 12 weeks.

The adherence rates were calculated for the last two blocks of the questionnaire, through the ratio between the total performed by the patient and the total prescribed by the health staff. Based on this assessment, patients were divided into two groups: good adherence (adherence equal or superior to 95%) and poor adherence (adherence lower than 95%). Aside from the PRAQ, the following three questionnaires were applied: *Critério de Classificação Econômica Brasil* (Brazilian Economic Classification Criteria) (CCEB),^13^ Pediatric Quality of Life Inventory (PedsQL),^14^ and Childhood Health Assessment Questionnaire (CHAQ).^15^

The demographic and clinical characteristics were described in absolute and relative frequencies, mean, standard deviation, and minimum and maximum values depending on the nature of the variables. The chi-square test and the Fisher exact test were used to compare categorical variables. To compare continuous variables, the Mann-Whitney test was used. Regarding the PRAQ, absolute and relative frequencies of the patients’ responses to the items of the questionnaire were evaluated. To evaluate redundancy between pairs of items within a block, the *Kappa* coefficient was calculated to check the level of agreement. For each of the five blocks of items, Cronbach’s alpha was evaluated as a measure of reliability (internal consistency).^16^

For the descriptive statistical analysis, means, medians, minimum, maximum, and standard deviation were calculated for all indexes. In addition, the Spearman correlation coefficient was evaluated to analyze the statistical correspondence between the percentage value for adherence and the total score of PRAQ and each index. A value equal or superior to 95% was adopted to indicate good adherence. The level of significance was set to be 5%. The statistic software Statistical Package for the Social Sciences (SPSS) 20 was used.

The study was approved by the local Research Ethics Board.

## RESULTS

Thirty-three patients were consecutively included in the study. Six months after the completion of the PRAQ, the adherence was assessed by the quantitative questionnaire. Poor adherence to the global treatment was observed in seven (21.2%) patients, and poor adherence to the medical treatment was detected in eight patients (24.2%). No differences between the groups were observed for any of the factors evaluated, except that females were more adherent than males. At the time of assessment, the following medications were being used: 21 (63.6%) methotrexate, 19 (57.6%) prednisone, eight (24.2%) hydroxychloroquine, seven (21.2%) azathioprine, three (9.1%) immunobiological, two (6.1%) cyclosporine, one (3%) leflunomide and one (3%) cyclophosphamide. Demographic, socioeconomic and clinical data of patients related to global adherence are shown in Table 1.

**Table 1:** Demographic and clinical data of patients related to global adherence (total n=33).

	Good adherence (n=26)	Poor adherence (n=7)	p-value
Female	20	2	0.02
Age at the PRAQ in years	10.7±3.8	9.2±5.7	0.39
Adolescents (≥12 years old)	14	3	0.68
Diagnosis			0.41
Juvenile idiopathic arthritis	9	4	
Systemic lupus erythematosus	8	2	
Dermatomyositis/polimyositis	7	0	
Local scleroderma	2	1	
Disease duration since diagnosis in months (mean±SD)	2.8 (±1.2)	3 (±1)	0,81
Active disease	16	4	1
PedsQL parents (n=26)	72.6±20.0	84.2±0.7	0.4
PedsQL patients (n=22)	76.9±15.1	69.7±8.6	0.22
CHAQ (n=26) (median, min-max)	0 (0-2)	0 (0-1)	0.75
Mother’s education level (n=31)			0.75
Middle school uncompleted	9	3	
Middle school completed	6	1	
High school completed	7	3	
College completed	2	0	
Number of people in the home	4.4±1.1	4.3±1.1	0.87
Number of children in the home	2.1±0.6	1.7±0.7	0.27
CCEB (n=28)			0.6
Class B	2	0	
Class C	19	5	
Class D	2	0	

PRAQ: Pediatric Rheumatology Adherence Questionnaire; PedsQL: Pediatric Quality of Life Inventory; CHAQ: Childhood Health Assessment Questionnaire; CCEB: *Critério de Classificação Econômica Brasil* (Brazilian Economic Classification Criteria); SD: standard deviation.

The data from the patient’s adherence related to the quantitative questionnaire - global adherence, medical adherence, and non-medical adherence (visits, exams, physical therapy, and orthosis) - are shown in Table 2.

**Table 2:** Percentage of adherence types for patients with good and poor adherence.

	Total	Good adherence	Poor adherence
Global adherence	33	26 (78.8%)	7 (21.2%)
Medical adherence	33	25 (75.8%)	8 (24.2%)
Visits adherence	33	32 (97.0%)	1 (3.0%)
Inter-visits adherence*	24	23 (95.8%)	1 (4.2%)
Physical therapy adherence	6	4 (66.7%)	2 (33.3%)
Orthosis adherence	5	3 (60.0%)	2 (40.0%)
Exams adherence	33	31 (93.9%)	2 (6.1%)

*Other specialties.

A new PRAQ questionnaire with 25 of the 46 original questions was generated as a result of the reliability analysis. Therefore, a final 25 questions were selected for the statistical data analysis. The final version of the questionnaire is shown in Table 3.

**Table 3:** Final version of the Pediatric Rheumatology Adherence Questionnaire.

Socioeconomic indexes
Q1. Is the distance between your home and the hospital/outpatient service prejudicial to your child’s treatment?
Q2. Is the means of transportation used to attend the visits and exams prejudicial to the treatment?
Q3. Is the number of children you have prejudicial to your sick child’s treatment?
Q4. Is your work in the home or profession prejudicial to your child’s treatment?
Q5. Are visits and exams prejudicial to your work?
Indexes related to the healthcare team and system
Q6. Is it difficult to understand the explanations given by the doctor?
Q7. Does the doctor provide too little information about your child’s condition?
Q8. Does the doctor provide too little information on the medication your child is taking?
Indexes related to the health condition
Q9. Do you think your child is healthy?
Q10. Do you think your child’s complaints are sometimes exaggerated?
Q11. Do you think that despite their condition, your child has a good quality of life?
Q12. Do you think that despite your child’s condition, your family has a good quality of life?
Indexes related to the therapy
Q13. Is your child refusing or has refused any medication?
Q14. Does/Did your child have any kind of reaction to a medication?
Q15. Is the number of pills your child is taking prejudicial to the treatment?
Q16. Is the type of medication (tablet, intravenous, subcutaneous etc.) prejudicial to your child’s treatment?
Q17. Is the dosage schedule of the medication prescribed by the doctor a problem for your child’s treatment?
Q18. Do you allow your child to take his/her medication without the assistance of an adult?
Q19. Have you forgotten to give your child his/her medication?
Q20. Have you failed to give medication to your child because he/she refused it?
Q21. Have you failed to give medication to your child because he/she was not feeling well?
Q22. Do you think your child does not need the medication prescribed?
Q23. Do you have doubts about the medication your child is taking?
Indexes related to the patient/caregiver
Q24. Do you have relationship problems with your child?
Q25. Do you think you are wasting your time with your child’s treatment?

No correlation was observed between the PRAQ scores and the percentages of adherence (p=0.763 for global adherence and p=0.46 for medical adherence), as well as the stratification for each index, except for a tendency to a correlation between socioeconomic index and poor adherence to medical treatment (p=0.08) (Table 4).

**Table 4:** Spearman correlation between indexes and medical adherence.

Socioeconomic factors	-0.335 (p=0.0873)
Factors related to healthcare team and system	0.108 (p=0.5552)
Factors related to health condition	0.248 (p=0.1645)
Factors related to therapy	-0.008 (p=0.9696)
Factors related to patient and caregiver	-0.080 (p=0.9632)

## DISCUSSION

This study proposed to develop a questionnaire that could allow the early detection of patients at risk for poor adherence to medical and non-medical treatment in children and adolescents with chronic rheumatic diseases, and several indexes were evaluated corresponding to adherence to medical and non-medical treatment. Regarding the indexes evaluated by the PRAQ questionnaire, we found a tendency to a negative correlation between socioeconomic index and poor adherence to medical treatment. This may mean that patients who had higher scores in socioeconomic index may be at risk for progression to poor adherence to medical treatment. Studies evaluating adherence to treatment in chronic diseases report several factors related to poor adherence and different degrees of association among them, including socioeconomic factors, like financial difficulties.^17^ ^,^ ^18^

Healthcare professionals often neglect the meaning of poorly-adherent patients, despite the negative consequences on healthcare and cost.^19^ The use of questionnaires to evaluate the adherence of patients to treatment is very useful particularly in patients with recently diagnosed chronic diseases. In these situations, good adherence may provide a better response to treatment and improve the prognosis and long-term quality of life.

Civita et al. developed questionnaires validated in English and French.^7^ These questionnaires focused on the evaluation of the adherence to the JIA treatment. They comprise the Parent Adherence Report Questionnaire (PARQ), which evaluates adherence from a caregiver’s point of view and specific adherence questions, such as the difficulty in following the treatment, frequency of negative reactions, and the level of efficacy of treatment.^7^ PARQ consists of four questions repeated for every component of the treatment, including the use of drugs, orthosis, and the practice of physical activities. In a recent review, we accentuated the need for an identification of factors that interfere with the adherence to the treatment in JIA patients, as well as the means to evaluate this adherence in addition to actions that can be taken to minimize the consequences of poor adherence.^20^

In the past, questionnaires have already been developed to evaluate adherence and to improve the monitoring of the treatment. Recently, Hughes et al. published a short version of the “Compliance Questionnaire for Rheumatology” with good reliability and validity. The concise arrangement of questions provided better usability in the clinical examination of the patient.^21^ So far, in the pediatric age group, questionnaires for the evaluation of adherence were mostly related to a specific disease such as diabetes, asthma, epilepsy, and AIDS.^22^ ^,^ ^23^ ^,^ ^24^ ^,^ ^25^

Despite an overestimation of adherence rates, the self-management questionnaires are considered a good tool for clinical settings because they are easy to apply and they allow for the evaluation of attitudes, intentions, and behaviors. An additional advantage is that questionnaires can help to find out how and why a patient is not adherent. This enables objective interventions matched to the specific situation of each patient.^21^ ^,^ ^26^ ^,^ ^27^

This study has some limitations. The power of the study is very low due to few numbers of patients. This fact limits the discrimination of the patients in respect to diagnosis and also limits the chance to compare our findings to other adherence score systems. Chronic rheumatic diseases in children have a relatively rare incidence, and the inclusion criteria were restricted to recent diagnostic situations, which may have impaired the formation of the sample. A multicenter study or a longer study may be needed to give the sample a greater power to evaluate the questionnaire.

Regarding the structure of the PRAQ, there is an imbalance in the number of items attributed to each adherence factor which may lead to a misrepresentation, and some questions may lead to biased responses. Reviewing the structure of the questionnaire and reshaping some of the questions may result in better internal consistency. Recently, the PARQ questionnaire, that evaluates the adherence to the JIA treatment, was transformed to improve its psychometric properties.^28^

Another limitation is that the caregivers, not the adolescents, responded to the questionnaire. It is well known and recognized that adolescents with chronic diseases have always had adherence problems with medication.^29^

The usefulness of PRAQ questionnaire in clinical practice should continue to be evaluated. Due to the importance of a tool for the early detection of rheumatic patients at risk of poor adherence to treatment, the new PRAQ questionnaire should be reviewed and applied in a larger study to better define its validity and reliability.

## References

[1] Rapoff MA (2006). Management of adherence and chronic rheumatic disease in children and adolescents. Best Pract Res Clin Rheumatol.

[2] McGrady ME, Hommel KA (2013). Medication adherence and health care utilization in pediatric chronic illness: a systematic review. Pediatrics.

[3] Kroll T, Barlow JH, Shaw K (1999). Treatment adherence in Juvenile Rheumatoid Arthritis - A review. Scand J Rheumatol.

[4] Bugni VM, Ozaki LS, Okamoto KY, Barbosa CM, Hilário MO, Len CA (2012). Factors associated with adherence to treatment in children and adolescents with chronic rheumatic diseases. J Pediatr (Rio J).

[5] Martin LR, Williams SL, Haskard KB, DiMatteo MR (2005). The challenge of patient adherence. Ther Clin Risk Manag.

[6] Feldman DE, De Civita M, Dobkin PL, Malleson PN, Meshefedjian G, Duffy CM (2007). Effects of adherence to treatment on short-term outcomes in children with juvenile idiopathic arthritis. Arthritis Rheum.

[7] Civita M, Dobkin PL, Ehrmann-Feldman D, Karp I, Duffy CM (2005). Development and preliminary reproducibility and validity of the parent adherence report questionnaire: a measure of adherence in juvenile idiopathic arthritis. J Clin Psychol Med Settings.

[8] Petty RE, Southwood TR, Baum J, Bhettay E, Glass DN, Manners P (1998). Revision of the proposed classification criteria for juvenile idiopathic arthritis: Durban 1997. J Rheumatol.

[9] Petty RE, Southwood TR, Manners P, Baum J, Glass DN, Goldenberg J (2004). International League of Associations for Rheumatology classification of juvenile idiopathic arthritis: second revision, Edmonton, 2001. J Rheumatol.

[10] Hochberg MC (1997). Updating the American College of Rheumatology revised criteria for the classification of systemic lupus erythematosus. Arthritis Rheum.

[11] Bohan A, Peter JB (1975). Polymyositis and dermatomyositis. N Engl J Med.

[12] Cassidy JT, Petty RE (2005). Textbook of pediatric rheumatology.

[13] Associação Brasileira de Empresas de Pesquisa (ABEP) (2008). Dados com base no Levantamento Sócio Econômico - 2005 - IBOPE.

[14] Varni JW, Seid M, Kurtin PS (2001). PedsQL 4.0: reliability and validity of the Pediatric Quality of Life Inventory TM Version 4.0 generic core scales in healthy and patient populations. Med Care.

[15] Machado CS, Ruperto N, Silva CH, Ferriani VP, Roscoe I, Campos LM (2001). The Brazilian version of the Childhood Health Assessment Questionnaire (CHAQ) and the Child Health Questionnaire (CHQ). Clin Exp Rheumatol.

[16] Cronbach LJ (1951). Coefficient alpha and the internal structure of tests. Psychometrika.

[17] World Health Organization (2003). Adherence to long-term therapies: evidence for action.

[18] Pelajo CF, Sgarlat CM, Lopez-Benitez JM, Oliveira SK, Rodrigues MC, Sztajnbok FR (2012). Adherence to methotrexate in juvenile idiopathic arthritis. Rheumatol Int.

[19] Patel UD, Davis MM (2006). Physicians' attitudes and practices regarding adherence to medical regimens by patients with chronic illness. Clin Pediatr (Phila).

[20] Len CA, Miotto e Silva VB, Terreri MT (2014). Importance of adherence in the outcome of juvenile idiopathic arthritis. Curr Rheumatol Rep.

[21] Hughes LD, Done J, Young A (2013). A 5 item version of the Adherence Questionnaire for Rheumatology (CQR5) successfully: identifies low adherence to DMARDs. BMC Musculoskelet Disord.

[22] Harris MA, Wysocki T, Sadler M, Wilkinson K, Harvey LM, Buckloh LM (2000). Validation of a structured interview for the assessment of diabetes self-management. Diabetes Care.

[23] Skinner EA, Diette GB, Algatt-Bergstrom PJ, Nguyen TT, Clark RD, Markson LE (2004). The Asthma Therapy Assessment Questionnaire (ATAQ) for children and adolescents. Dis Manag.

[24] Modi AC, Monahan S, Daniels D, Glauser TA (2010). Development and validation of the Pediatric Epilepsy Medication Self-Management Questionnaire. Epilepsy Behav.

[25] Van Dyke RB, Lee S, Johnson GM, Wiznia A, Mohan K, Stanley K (2002). Reported adherence as a determinant of response to highly active antiretroviral therapy in children who have human immunodeficiency virus infection. Pediatrics.

[26] Hommel KA, Davis CM, Baldassano RN (2008). Medication adherence and quality of life in pediatric inflammatory bowel disease. J Pediatr Psychol.

[27] Rapoff MA (2002). Assessing and enhancing adherence to medical regimens for juvenile rheumatoid arthritis. Pediatr Ann.

[28] Toupin April K, Higgins J, Ehrmann Feldman D (2016). Application of Rasch analysis to the parent adherence report questionnaire in juvenile idiopathic arthritis. Pediatr Rheumatol Online J.

[29] No-referred authorship (2016). Can we improve adolescent adherence?. Drug Ther Bull.

